# Machine learning-based assessment of regional-scale variation of landslide susceptibility in central Vietnam

**DOI:** 10.1371/journal.pone.0308494

**Published:** 2024-10-25

**Authors:** Raja Das, Pham Van Tien, Karl W. Wegmann, Madhumita Chakraborty

**Affiliations:** 1 Center for Geospatial Analytics, North Carolina State University, Raleigh, North Carolina, United States of America; 2 Department of Marine, Earth, and Atmospheric Sciences, North Carolina State University, Raleigh, North Carolina, United States of America; 3 Vietnam Academy of Science and Technology, Institute of Geological Sciences, Hanoi, Vietnam; 4 Department of Earth and Environmental Geoscience, Washington and Lee University, Lexington, Virginia, United States of America; National Central University, TAIWAN

## Abstract

Recurrent landslide events triggered by typhoons and tropical storms over Vietnam pose a longstanding threat to the nation’s population and infrastructure. Changes in hydroclimatic conditions, especially the growing intensity and frequency of storms, have elevated landslide susceptibility in many parts of the country. This research examines the spatio-temporal variations in landslide susceptibility across central Vietnam over several years, using multi-temporal landslide inventories from Typhoon Ketsana (2009), Tropical Storm Podul (2013), and Typhoon Molave (2020). Additionally, the research explores the impact of individual landslide causative factors on the probabilistic occurrences of landslides. The post-event landslide susceptibility models of these three climate extreme events were developed using nine causative factors and a Random Forest machine learning algorithm. The results indicate a notable areal expansion of high to very high landslide susceptibility in the northern and eastern regions and a moderate reduction in the central and southern areas during the post-Molave period compared to the post-Ketsana period. These changes may be early indicators of increasing landslide susceptibility in response to changing hydro-climatic conditions. The research found that annual average rainfall and topographic elevation are the two most important variables influencing landslide prediction, showing a nonlinear relationship with landslide probability. The landslide susceptibility models achieved high Area Under the Receiver Operating Characteristic Curve (AUC) (>95%), accuracy (>89%), and sensitivity (>90%) scores, signifying the robustness of the models. Additionally, the uncertainty of the models was quantified and spatially mapped. This multi-temporal analysis of landslide susceptibility is crucial for understanding the regional susceptibility trends and identifying areas with increasing, decreasing, and consistently high susceptibility to landslides. These insights are invaluable for prioritizing mitigation and risk reduction strategies in landslide-prone regions and guiding appropriate land use planning.

## Introduction

Landslides pose a significant hazard to millions living in mountainous regions worldwide. Studies reported more than over 150,000 landslide fatalities between 1995 and 2014, while report 55,997 landslide-related deaths between 2004 through 2016 [1, 2]. With the global population exceeding 8 billion, the pressure to build housing and infrastructure in marginal areas prone to landslides is rising. In addition, heavy and prolonged precipitation is the leading cause of most fatal landslides. The frequency of rainfall-induced landslides are increasing due to the rise in extreme rainfall events resulting from human-caused global warming [3, 4]. According to the International Disaster Database (EM-DAT), between 1990 and 2015, landslides accounted for 1.3% of natural disaster fatalities, of which 1.3% of the fatal landslide events occurred in Asia [5].

Identifying the potential zones of future landslides remains the primary yet challenging task to attenuate the adverse impact of such occurrences especially in mountainous regions. Landslides are complex geological phenomena influenced by combinations of predisposing factors, such as topography, geology, hydrology and also known as geo-factors, with varying degrees of importance and triggered by external forces such as rainfall or earthquakes [6]. Therefore, understanding the interplay between predisposing factors and the occurrence of past landslides can provide valuable insights into the likelihood of future landslide occurrences. Landslide susceptibility assessment (LSA) is a crucial analytical technique that capitalizes on the interrelationship between various geo-factors and past landslides to predict the spatial probability of future landslides. LSA operates on the fundamental assumption that the same geo-factors that caused past landslides are likely to cause similar landslides in the future under comparable environmental conditions [7–9].

A plethora of literature is available on the various techniques for developing landslide susceptibility maps,generally divided into knowledge-driven (heuristic) and empirical (data-driven) approaches. Knowledge-driven methods consider the interrelationship between landslides and geo-factors as direct evidence for landsliding, and the interpretation of such relationships is quantified or qualitatively made by an expert to generate the landslide susceptibility map. However, such an approach often introduces subjectivity into the model [6, 10]. In contrast, data-driven methods rely on the statistical or mathematical relationship between observed landslides and geo-factors, which generally leads to more accurate predictions than knowledge-driven approaches [11, 12]. Recently, machine-learning algorithms have gained significant popularity in landslide susceptibility analysis due to their capability to process large amount of complex nonlinear information with greater accuracy than traditional data-driven methods. Various machine learning algorithms such as Artificial Neural Networks, Support Vector Machine, Random Forest, Naive Bayes, Boosted Regression Tree, and Logistic Regression have widely been applied to landslide susceptibility analysis to improve the models’ prediction accuracy [12–18].

The escalating incidence of fatal landslides worldwide is of concern, with a discernible upward trajectory [2]. Notably, mountainous regions in tropical countries are anticipated to witness a rise in the numbers of landslide incidences attributed to the escalating frequency of extreme precipitation events induced by intense typhoons and hurricanes [19]. Despite the heightened susceptibility to landslides and a significant number of fatalities in Southeast Asia, the climatic impact on landslide occurrences in this region has received limited attention in the existing literature [4]. Vietnam is one of the Southeast Asian countries severely affected by mass wasting with torrential rainfall associated with typhoons being the primary trigger for catastrophic landslides [20–25]. Vietnam’s central region is highly susceptible to landslides and extensive flooding, and landslides are caused by extreme weather events have resulted in significant damage and casualties in the region over the past several decades [21, 23, 26–28]. While prior studies have focused on developing landslide susceptibility maps in parts of central Vietnam [29, 30], these efforts have not adequately explored changes in the spatio-temporal patterns of landslide susceptibility resulting from land-falling typhoons in the region over the past several decades. Moreover, these studies relied on incomplete and non-time-stamped landslide data to develop susceptibility models, highlighting the need for a more comprehensive landslide susceptibility modeling utilizing complete landslide inventories. In this regard, this research seeks to: (i) develop landslide susceptibility models using the complete multi-temporal landslide inventories of Typhoon Ketsana (2009), Tropical Storm Podul (2013), and Typhoon Molave (2020) through the application of the Random Forest algorithm; (ii) investigate changes in spatio-temporal dynamics of landslide susceptibility in the region; (iii) understand the roles of different landslide causative factors or geo-factors in controlling the spatial disposition of landslides; and (iv) quantify the models’ uncertainty. By pursuing these objectives, this research endeavors to fill a critical gap in understanding spatio-temporal dynamics of landslide susceptibility and controlling geo-environmental factors for landslide initiation in parts of central Vietnam resulting in valuable insights for both academic knowledge and practical applications for regional landslide hazard mitigation and preparedness.

## Materials and methods

### Study area

**Fig 1:**
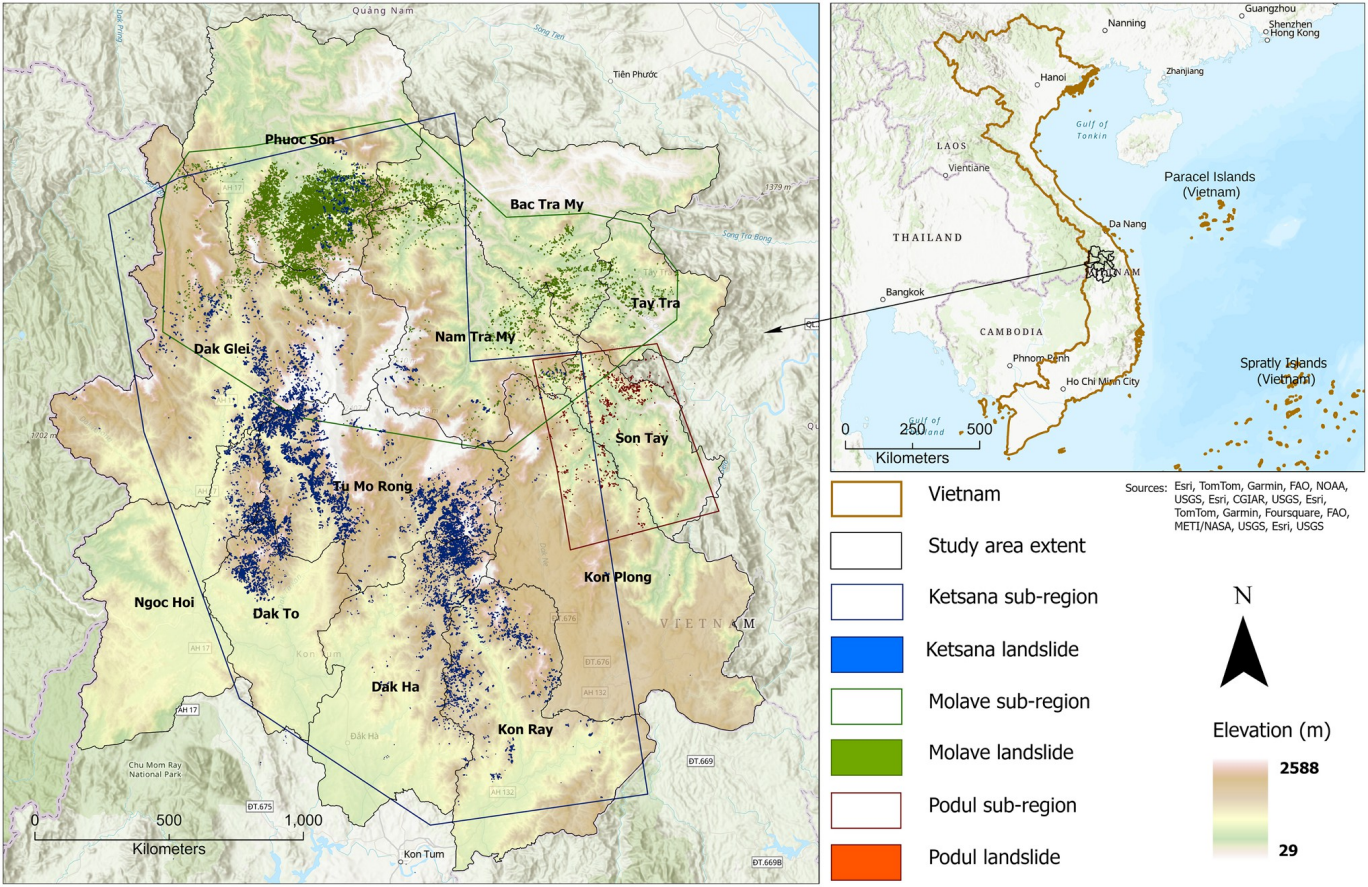
Geographic map of Vietnam (right) marked by the study area overlying the regional topography. Left: study area sub-regions, corresponding to the areas impacted by Typhoon Ketsana and Molave, and cyclone Podul, contain the color-coded landslide inventories used in the investigation. Source: Vietnam administrate boundaries are available in the Humanitarian Data Exchange website. DOI: 10.6084/m9.figshare.25710555.

The area of investigation covers approximately 10,409 km². It incorporates twelve administrative districts spread across three provinces, including seven districts of Kon Tum province of the Central Highland region (66% of the study area), three districts from the southern portions of Quang Nam, and two districts from the western reaches of Quang Ngai of the South-Central Coast region (Fig 1). Rugged mountains and deep valleys, with elevations ranging from 29 to 2,588 m above sea level are characteristic of the study area. High mountainous topography stretches NNE to SSW for 135 km across the study area. The elevation gradually decreases towards the northern and eastern parts of the Quang Nam and Quang Ngai Provinces, while wide valleys and flat terrains characterize the southern part. The study area receives a high amount of rainfall and the onset and duration of rainy season varies slightly among the provinces. The annual average rainfall in Kon Tum province varies from 1,700 mm to above 3,000 mm (rainy season lasts from April to November), whereas in Quang Nam annual average rainfall ranges from 2,020 to 4,000 mm (rainy season lasts from September to December), and in Quang Ngai it varies between 2,500 to 3,500 mm (rainy season lasts from August to November) [27, 30–32]. The study area is underlain by the Kon Tum Massif (KTM) tectonic unit, which is the largest Precambrian basement exposure in Indochina block and is comprised of Mesoproterozoic igneous and late Paleoproterozoic to late Neoproterozoic sedimentary rocks [33, 34]. The metasedimentary rocks of the KTM unit exhibit considerable variation in the degree of metamorphism ranging from low-grade (e.g., biotite schist, quartz mica schist) to high and ultrahigh temperature granulite facies rocks (e.g., granite genies, migmatite, Charnockite) [35]. Neogene to Quaternary sedimentary rocks such as conglomerate, sandstone, and siltstone are also abundant in the Kon Tum region [36]. Major faults such as the Po Ko and Dac To Kan faults cross the study area along with several other minor ones making the rock mass fragile and prone to mass wasting [37].

### Landslide data

Landslides triggered by Typhoon Ketsana (2009), Tropical Storm Podul (2013), and Typhoon Molave (2020) were identified from high-resolution satellite imagery (Fig 1). RapidEye satellite imagery (5m/pixel) was used to map landslides triggered by Ketsana and Podul. In contrast, PlanetScope satellite data (3m/pixel) was utilized to identify landslides triggered by Molave Table 1. The analysis-ready images were collected from Planet Labs [38] with the lowest possible cloud cover and the shortest post-event acquisition time. Landslides were initially identified through analysis of the difference in the Normalized Differential Vegetation Index (dNDVI) derived from pre- and post-event satellite imagery for the Podul and Molave events under the premise that decreases in NDVI values between images correspond to areas affected by landslides and concurrent flooding. Following this initial identification, the landslide mapping underwent manual refinement to eliminate areas affected by flooding and other non-landslide factors, resulting in the final landslide delineation. Conversely, given the absence of pre-event imagery for Ketsana, various thresholds of NDVI values were utilized to delineate landslides. Subsequently, these initially mapped landslides underwent manual refinement to enhance mapping precision and minimize errors. A total of 8,744 landslides were mapped for typhoon Ketsana, having a cumulative area of 51.39 *km*^2^ with a mean landslide size of 5,877 *m*^2^. In contrast, Tropical Storm Podul triggered lesser numbers of landslides, totaling 915, with a cumulative landslide area of 1.42 *km*^2^ and mean size of 1,556 *m*^2^. Typhoon Molave triggered a total of 10,257 landslides encompassing a cumulative area of 31.62 *km*^2^ with a mean landslide size of 3,082 *m*^2^. Triggered by torrential rainfall, these landslides predominantly began as shallow failures under complete saturation conditions of soil slopes and frequently merged to form debris flows under the presence of excess water on the slope.

**Table 1:** Summary of the satellite imagery used for landslide mapping.

Typhoon	Satellite	Spatial resolution	Date of pre-event image acquisition	Date of post-event image acquisition	Max cloud coverage
Ketsana	RapidEye	5m	Not available	Multiple images acquired between 12-26-2009 and 02-26-2010	<5%
Podul	RapidEye	5m	2-18-2013	1-8-2014	≤ 1%
Molave	PlanetScope	3m	02-03-2020 and 02-06-2020	12-29-2020 and 03-19-2021	≤ 1%

### Model variables

**Fig 2:**
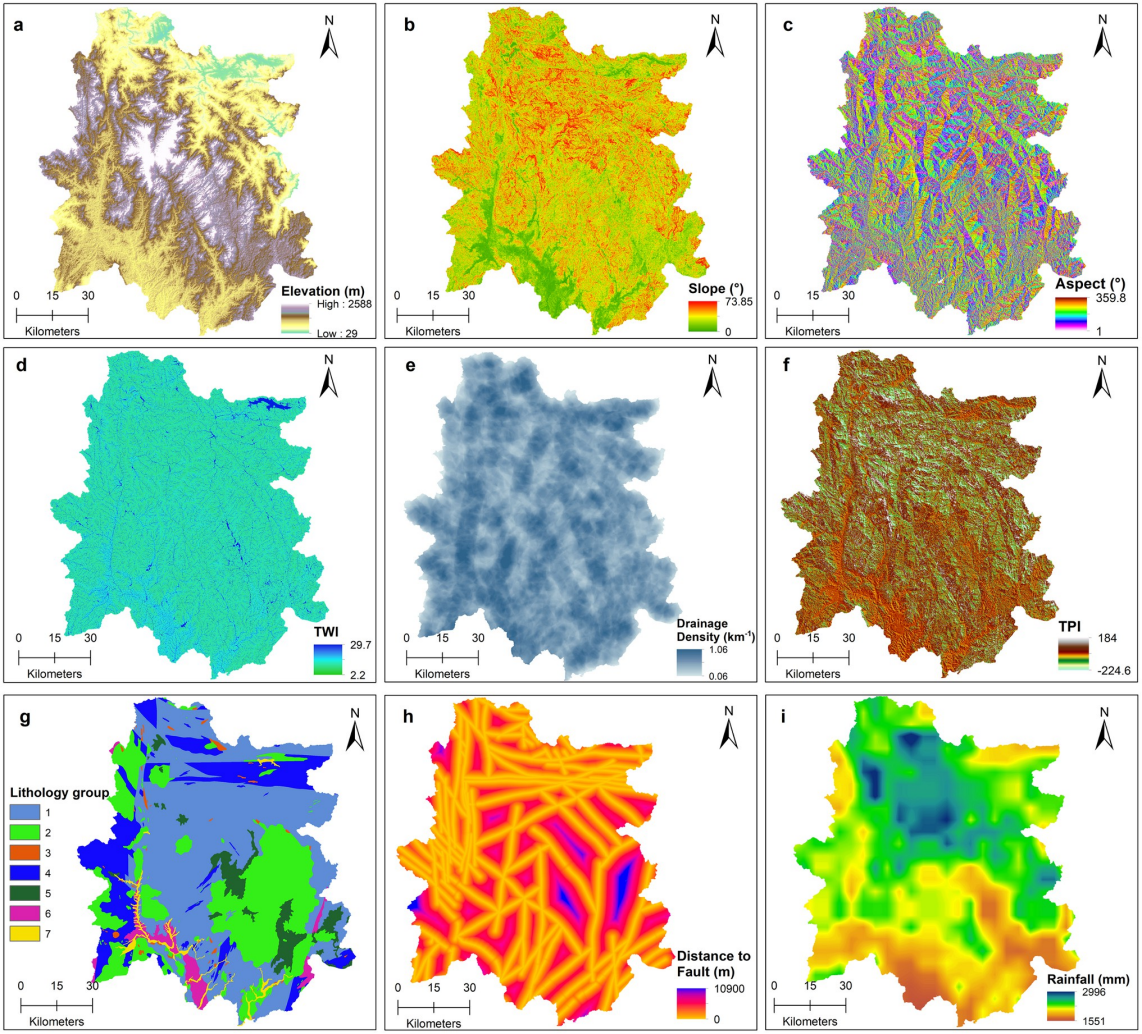
Variables (geo-factors) used in developing the Random Forest models. TWI and TPI are the Topographic Wetness and Position Indices, respectively. Rocky types (lithology) across the study are are differentiated as 1 = Mafic Metamorphic Rocks with quartz-poor component, 2 = Igneous—Intrusive Acid-to-Neutral Rocks, 3 = Igneous—Intrusive Mafic-to-Ultramafic Rocks, 4 = Felsic Metamorphic Rocks with quartz-rich component, 5 = Igneous- Extrusive Mafic-to-Ultramafic Rocks, 6 = Sedimentary Clastic Rocks, and 7 = Quaternary Sediments. Data source: The 30-meter NASADEM can be accessed from Earth Data. Elevation, Slope, Aspect, TWI, TPI, and Drainage density were derived from the NASADEM. Rainfall data was accessed from the website of Climate Hazards Group InfraRed Precipitation with Station data CHIRPS. The lithology and distance to fault data are included in the supplementary material (S2 Data). DOI: 10.6084/m9.figshare.25710138.

The study utilized nine explanatory variables or geo-factors for the development of landslide susceptibility models, namely, elevation, slope angle, aspect, Topographic Position Index (TPI), Topographic Wetness Index (TWI), distance to fault, drainage density, lithology, and annual average rainfall (Fig 2). A summary of the data source and their corresponding resolution is provided in Table 2. Elevation plays a crucial role in determining landslide susceptibility, as it significantly affects key elements such as local climate, vegetation coverage, soil composition, erosion rates, and water dynamics, all collectively contributing to slope stability. Variations in elevation can lead to changes in these critical components, thereby influencing the likelihood of landslides within a region. Slope angle significantly influences landslide initiation, with steeper slopes experiencing increased shear stresses due to gravity, elevating the risk of slope instability and potential landslide events. However, very steep slopes, often characterized by minimal soil cover and limited material accumulation, are predominantly susceptible to rockfalls. This contrasts with less steep slopes, where conditions are more conducive to shallow landslides or debris flows due to greater material accumulation and soil presence. The slope aspect can influence weathering, precipitation, moisture content, solar radiation, vegetarian type and density, which can influence the probability of landslides. A 30-meter NASADEM [39], an improved product of the Shuttle Radar Topography Mission (SRTM) digital elevation model (DEM), was used to generate the topographic variables.

**Table 2:** Summary of variables used in the development of landslide susceptibility models.

Variable	Resolution/Scale	Source
Elevation	30 meter	NASA Shuttle Radar Topography Mission DEM
Slope	30 meter	NASA Shuttle Radar Topography Mission DEM
Aspect	30 meter	NASA Shuttle Radar Topography Mission DEM
Topographic Wetness Index (TWI)	30 meter	NASA Shuttle Radar Topography Mission DEM
Topographic Wetness Index (TPI)	30 meter	NASA Shuttle Radar Topography Mission DEM
Drainage density	30 meter	NASA Shuttle Radar Topography Mission DEM
Lithology	1:200,000	Geological and Mineral Resources map
Distance to fault	1:200,000	Geological and Mineral Resources map
Rainfall	5566 meter	Climate Hazards Group InfraRed Precipitation with Station data (CHIRPS)

TWI is a hydrological variable that quantifies the effect of local topography on runoff flow direction and accumulation of water in a region and indicates the spatial variation of soil moisture and surface saturation. The TWI was derived from the DEM using the following formula:

$$\begin{array}{c} TWI=\text{ln}(\frac{A}{\text{tan}(\beta )}) \end{array}$$ (1)

where *A* is the upslope area per unit contour length, and *β* is the slope angle. The TWI value decreases with an increase in the slope gradient, and during rainfall, these low TWI regions become saturated with water, which leads to an increase in landslide susceptibility. High TWI values are associated with low slope gradients or flat areas, indicating zones of water accumulation, largely unfavorable for landslides. Drainage density can affect landslide susceptibility through several mechanisms. Slopes with higher drainage density experience increased erosion, destabilizing them and increasing their vulnerability to landslides. Additionally, higher drainage density enhances water availability on slopes, leading to elevated pore water pressure and greater saturation within the slope material. This increased saturation reduces the material’s shear strength, accelerating the likelihood of slope failures.

The Topographic Position Index (TPI) measures the difference between the elevation of a specific cell and the average elevation of surrounding cells within a designated radius. As a key geomorphological variable, positive TPI values indicate ridges or hills, while negative values signify valleys or topographic depressions. Although landslides can occur on ridges and steep slopes, TPI is valuable in landslide susceptibility mapping as it helps identify potential landslide-prone areas by distinguishing between various topographic features.

The lithological composition of an area plays a crucial role in determining the strength and cohesion of rock and soil properties that can directly impact the occurrence of landslides. Different lithological units have varying resistances to weathering and erosion, which can significantly affect slope stability. The lithology was reclassified into seven groups based on their compositions, relative weathering rate, and strength parameters (Table 3). Fault zones contain fractured formations and heavily weathered materials contributing to slope instability [30, 40]. Generally, areas further from these tectonic structures tend to experience fewer landslides due to reduced structural weakness [41]. The lithological and distance to fault maps were derived from Vietnam’s Geological and Mineral Resources map at 1:200,000 scale. Rainfall is a main landslide triggering parameter because it directly affects the soil saturation level, which is critical for landslide initiation [42, 43]. Prolonged or intense rainfall can increase the pore water pressure within the slope, reducing the soil and rock strength, facilitating landslide initiation. Annual average precipitation was derived from the Climate Hazards Group InfraRed Precipitation with Station data (CHIRPS) [44] for the rainy season, i.e., July to November from 1990 until the year of the landslide event. For example, in the case of the typhoon Ketsana-induced landslide susceptibility model, annual average rainfall data from 1990 to 2009 was incorporated. Similarly, for the post-Molave landslide susceptibility model, rainfall information spanning from 1990 to 2020 was integrated into the model.

**Table 3:** Reclassified lithological units in the study area.

Group	Description	Area(%)
1	Mafic Metamorphic Rocks with quartz-poor component	46.9
2	Igneous—Instrusive Acid-to-Neutral Rocks	26.6
3	Igneous—Intrusive Mafic-to-Ultramafic Rocks	0.8
4	Felsic Metamorphic Rocks with quartz-rich component	16.1
5	Igneous- Extrusive Mafic-to-Ultramafic Rocks	5.0
6	Sedimentary Clastic Rocks	2.8
7	Quaternary Sediments	1.8

### Modeling algorithm

Development of the landslide susceptibility models utilized the Random Forest (RF) machine learning algorithm, an ensemble-based, non-parametric approach consisting of multiple individual decision trees (DT). Each DT consists of several nodes, each representing a variable used in the model. The model-building process starts with the root node and gradually splits into decision nodes selected based on the Gini Impurity score. This score calculates the probability of misclassifying a randomly selected observation by a predictor variable in the data set. The variable with the lowest Gini impurity rises to the top of the tree as the root node for further splitting. This process repeats until no more splits are possible, which results in a terminal node.

The RF model uses a ‘Bagging’ technique, combining bootstrap and aggregation. In Bagging, a random subset of data from the training set is selected with replacement, allowing multiple selection of the same record/row of the training dataset for the construction of a DT. The RF model leverages bootstrapping to generate multiple uncorrelated DTs, which are trained parallelly on independent bootstrap data samples. Approximately two-thirds of the training data records go into the construction of the DTs, while one-third of the data are used for model validation by calculating the out-of-bag error of the RF model. Subsequently, the model utilizes the ‘aggregation’ technique to generate the final prediction by taking the majority vote (for classification) or all DTs’ average prediction (for regression). A single DT is a weak learner; however, aggregating weak learners tends to form a strong learner, resulting in better model performance. Since RF models use multiple independent DTs (weak learners) and aggregate individual predictions to determine the model’s outcome, the variance in the RF model significantly decreases compared to a single DT that often suffers overfitting, resulting in a low bias-high variance model.

### Landslide susceptibility model development

Three landslide susceptibility models were created employing a RF algorithm, utilizing landslide inventories from Ketsana, Podul, and Molave at a 75:25 ratio for training and testing purposes. To develop the landslide susceptibility models, landslide points were systematically generated within the polygons, ensuring that each polygon contained at least one point. The number of landslide points within a polygon corresponded to the size of the polygon, with larger landslide areas accommodating more points. Conversely, non-landslide points were randomly generated at a minimum distance of 100 meters from the landslide polygons. The random points were generated using ArcGIS software. Before generating random points within the landslide polygons, these polygons were partitioned into training and testing datasets. This step was crucial to prevent landslide points from the same landslide being included in both datasets, which could artificially inflate the accuracy metrics of the model. This approach helped ensure the integrity of the training and testing datasets, facilitating the development of a robust RF model for landslide susceptibility assessment. While generating the landslide susceptibility models, landslide information from previous events was retained and cumulatively added into the next landslide susceptibility model. The first susceptibility model utilized landslide information from typhoon Ketsana, while the second model incorporated data from both Ketsana and Podul. Similarly, the final landslide susceptibility map integrated data from Ketsana, Podul, and Molave-triggered landslides. This additive sample selection technique was implemented to ensure that information on landslide susceptibility from previous models is retained and transferred to subsequent models, thereby more effectively characterizing the changes in spatial and temporal patterns of landslide susceptibility over time. For the post-Ketsana susceptibility model, a total of 21,596 landslide points and 20,687 non-landslide points were utilized for model training, with 5,234 landslide points and 4,858 non-landslide points used for model validation. Similarly, for the post-Podul susceptibility model, 23,627 landslide points and 20,687 non-landslide points were used for training, and 6,110 landslide points and 4,858 non-landslide points were utilized for validation. In the case of the post-Molave model, 49,111 landslide points and 46,445 non-landslide points were employed for training, and 16,781 landslide points and 14,887 non-landslide points were used for validation.

The RF model was built using R statistical software. A five-fold cross-validation was applied to the RF model and repeated five times. Mtry, a hyperparameter of the RF model, was selected to optimize the model. Mtry indicates the number of randomly selected variables available for splitting at each decision tree node. The optimum numbers of Mtry used in the final model were selected based on the highest accuracy score achieved during model training. The number of decision trees was fixed at 500. This process was repeated three times, incorporating landslide information from different landslide inventories to generate the landslide susceptibility models.

### Model validation and uncertainty

The landslide susceptibility models were evaluated using various matrices on test data including: (i) Area under the Receiver Operating Characteristic (ROC) Curve (AUC), (ii) Accuracy of the model that indicates the fraction of correctly classified samples, (iii) Sensitivity and Specificity, which are the proportion of actual positive (landslide) and negative(non-landslides) classes that are correctly identified by the model, respectively, and (iv) Out-of-bag error (OBB), which is obtained during the generation of the RF model on training data and indicates an error in classifying the positive (landslide) and negative(non-landslides) during model training. The uncertainty associated with the RF model was quantified at a pixel-by-pixel level by calculating the standard deviation of predictions from individual trees for each corresponding pixel [45].

## Results

### Landslide susceptibility

**Fig 3:**
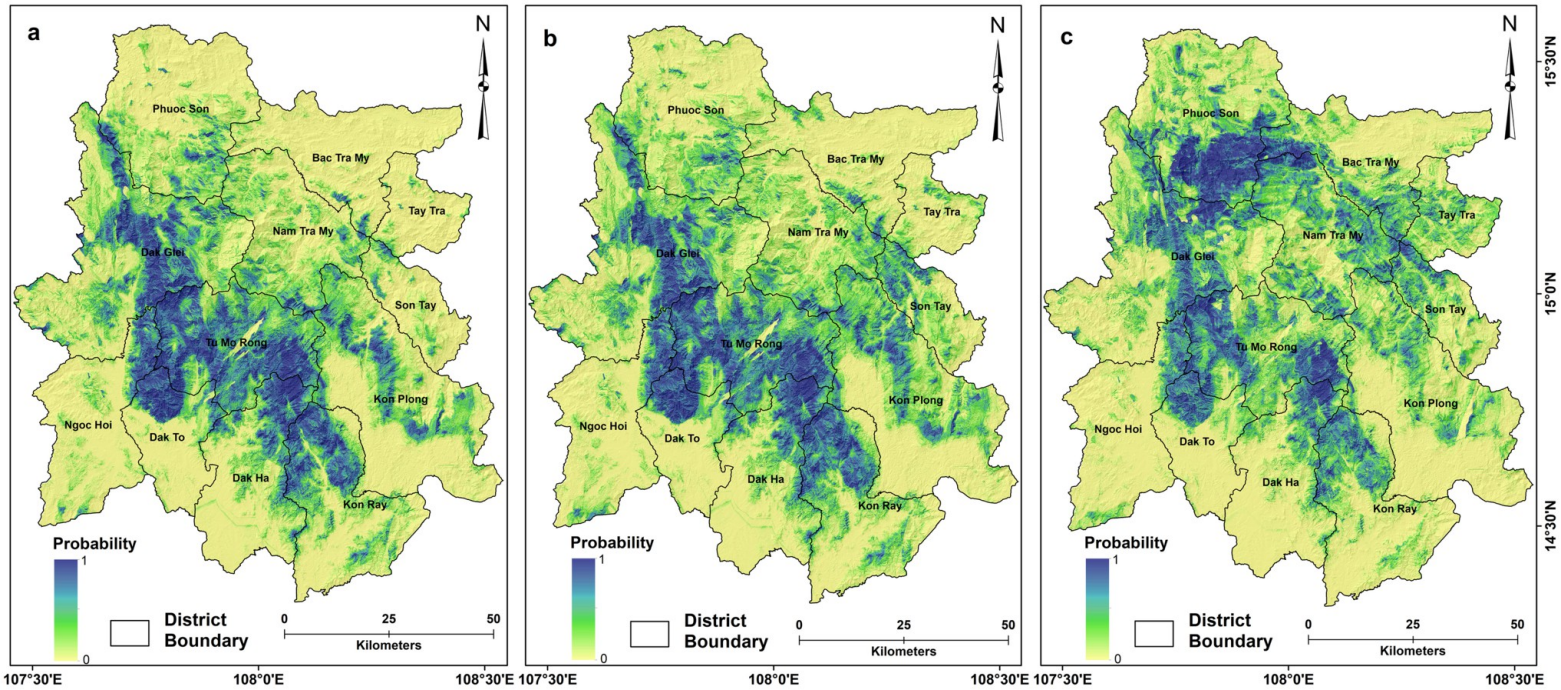
Landslide susceptibility maps of the study area were developed using the RF algorithm for (a) post-Ketsana, (b) post-Podul, and (c) post-Molave periods. A gradual increase in landslide susceptibility is evident after each typhoon/storm event in the past decades. Data Source: District boundary, https://cmr.earthdata.nasa.gov, https://www.chc.ucsb.edu/data/chirps, Institute of Geological Sciences, (VAST). DOI: 10.6084/m9.figshare.25710573.

Three landslide susceptibility maps were generated for the study area for three different periods: post-Ketsana (2009), post-Podul (2013), and post-Molave (2020) (Fig 3a–3c). The results revealed significant changes in landslide susceptibility across the study area over time. The post-Ketsana susceptibility map showed the central and southeastern parts of the study area to be very highly susceptible, while susceptibility was moderate to low in the northern parts and low in other areas. The model classified 54.87% of the study area as having very low landslide susceptibility, while 8.94% and 10% of the area are high to very highly prone to landslides.

**Fig 4:**
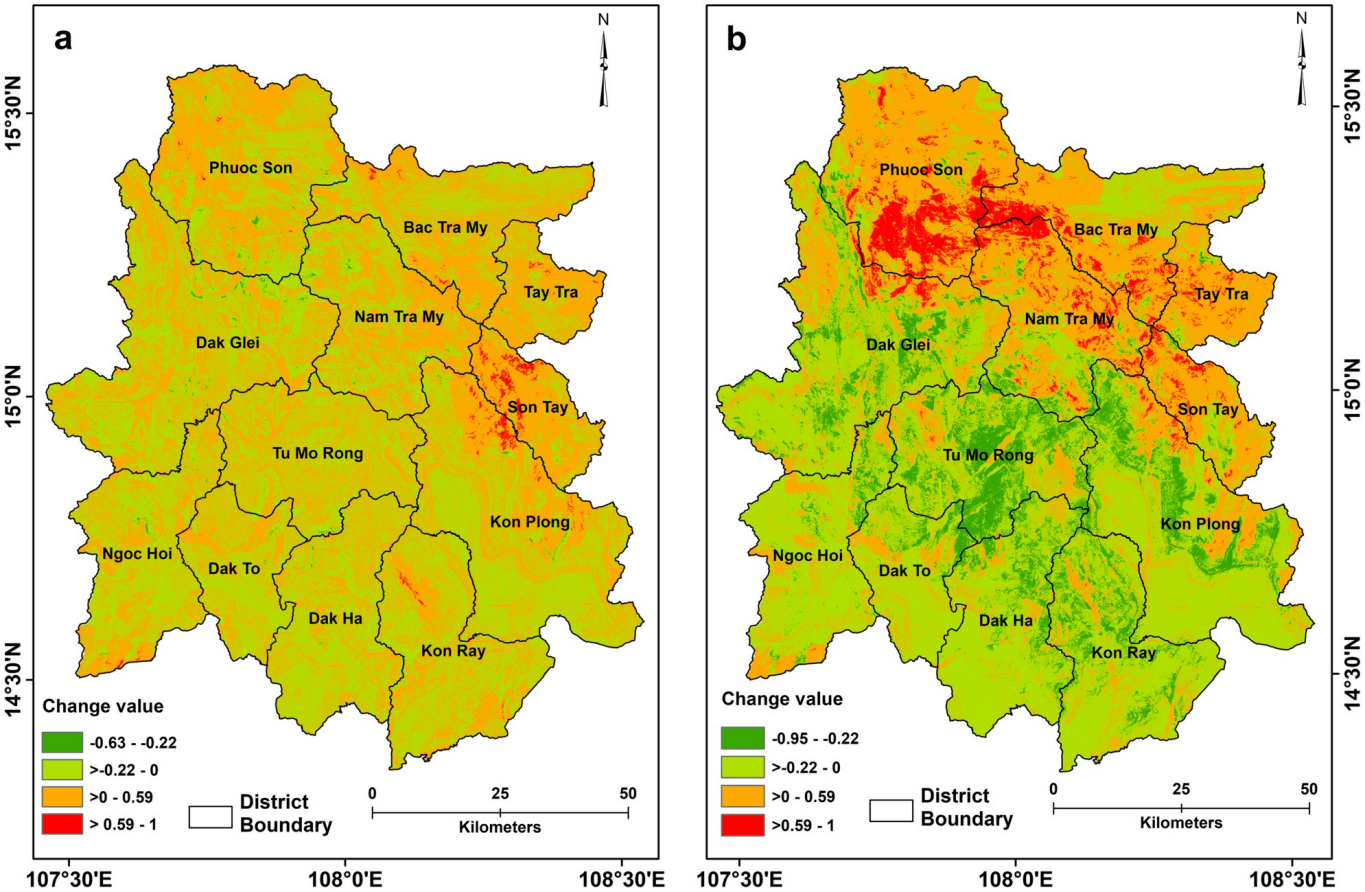
Maps illustrating the absolute change in probabilistic landslide susceptibility values following the impact of (a) Storm Podul and (b) Typhoon Molave in comparison to the post-Ketsana susceptibility conditions. Positive values denote an increase in landslide susceptibility, while negative values indicate a decrease in susceptibility compared to the post-Ketsana condition. District boundary, https://cmr.earthdata.nasa.gov, https://www.chc.ucsb.edu/data/chirps, Institute of Geological Sciences, (VAST). DOI: 10.6084/m9.figshare.25710582.

The eastern part, which had low susceptibility to landslides during the post-Ketsana period, exhibited an increase in susceptibility in the post-Podul susceptibility model (Fig 4a). Approximately 0.5% of the study area observed a substantial rise in susceptibility values and 51.75% experiencing a low to moderate increase in susceptibility values compared to the post-Ketsana scenario. While 0.25% area experienced a high drop and 47.5% area encountered a low to moderate drop in susceptibility compared to the post-Ketsana landslide susceptibility values. The post-Podul landslide susceptibility scenario showed that 50.27% of the area had very low susceptibility, while high to very high landslide susceptibility zones accounted for 9.68% and 10.48% of the study area, respectively. The final landslide susceptibility model, i.e., the post-Molave model (Fig 3c) showed a significant increase in landslide susceptibility in the entire northern region comprising Phuoc Son, Bac Tra My, and Nam Tra My districts of Quang Nam province and Tay Tra district of Quang Ngai province. These regions were largely low in landslide susceptibility in the post-Ketsana and post-Podul models (Fig 4b). However, increased landslide activity in this region during the Molave event resulted in an enormous increase in landslide susceptibility. Approximately 4.58% and 36.55% of the study area witnessed a high and moderate increase in landslide susceptibility values,respectively, compared to the post-Ketsana scenario. Interestingly, there was a decrease in the probability of landslides in the central to southern region. Around 10.71% of the area exhibited a high plunge, while 48.15% experienced a low to moderate descent in susceptibility values compared to the post-Ketsana landslide susceptibility conditions. Nevertheless, the overall probability of landslides remains very high in this region. The post-Ketsana model showed that 10.35% and 10.05% of the study area are high to very highly prone to landslides, while 49.35% of the area qualified as having very low susceptibility. The districts located in the southern part of the study area, such as Ngoc Hoi, Dak Ha, and Kon Ray of Kon Tum province largely remained low to very low in landslide susceptibility throughout the investigation period, owing to their lower topographic altitude and relatively gentle slopes unfavorable for landslide initiation. However, portions of these districts, especially those adjacent to the central part of the study area, remained highly susceptible to landslides in the investigation period.

### Role of landslide causative factors

**Fig 5:**
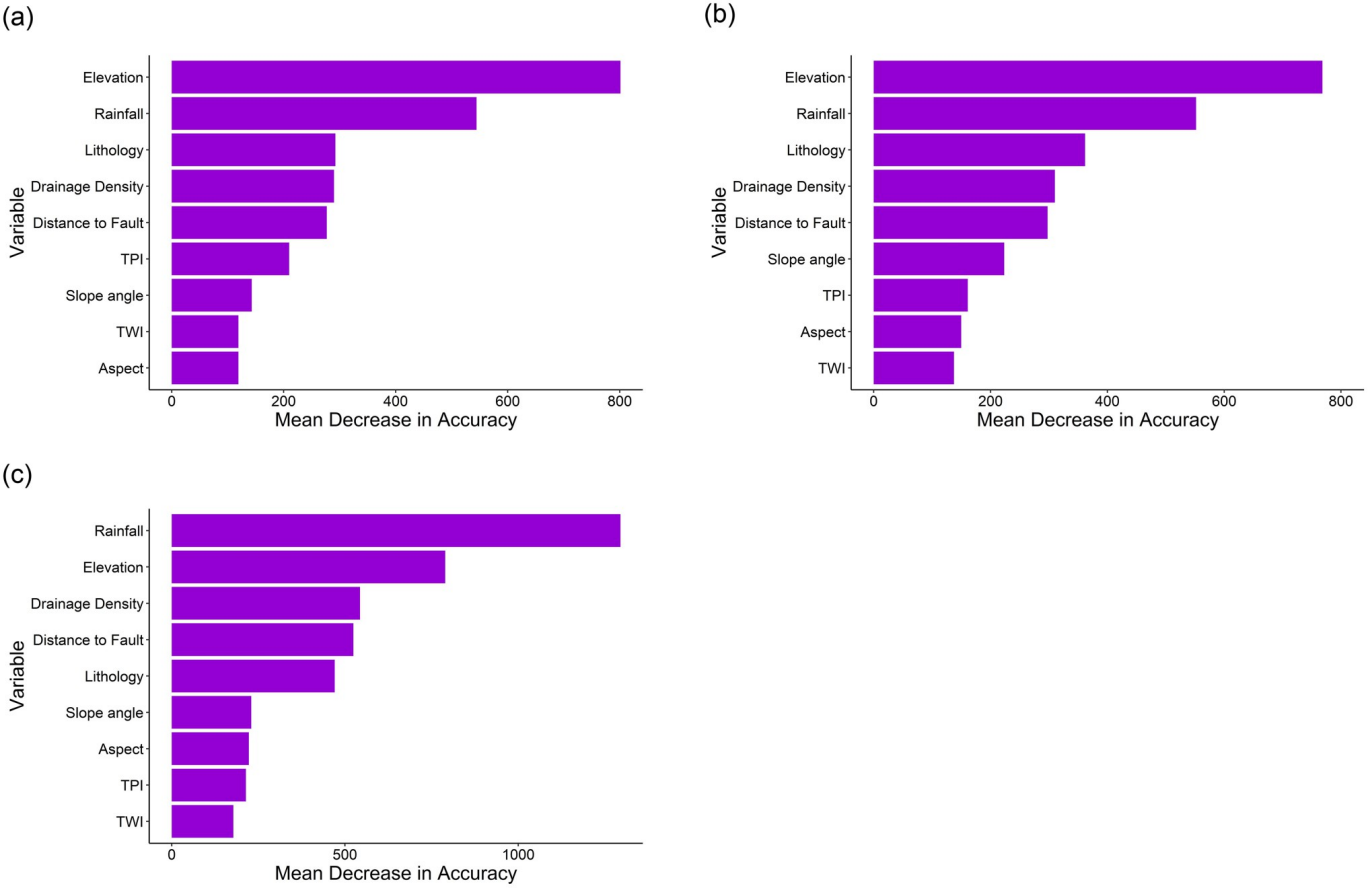
Plots showing Mean Decrease in Accuracy of the three landslide susceptibility models developed using the RF algorithm for the (a) post-Ketsana, (b) post-Podul, and (c) post-Molave periods. The plots show the relative importance of different variables in predicting landslide susceptibility in each period.

Fig 5a–5c display the Mean Decrease in Accuracy (MDA) for the three landslide susceptibility models. The MDA quantifies the loss in the model’s accuracy by excluding a variable. The higher the accuracy loss, the greater the variable’s importance in aiding accurate predictions. The results indicate that rainfall, elevation, and lithology are the three most important variables for predicting landslides. In addition, distance from faults and drainage density round out the top five variables. Variable importance refers to the relative contribution of each input variable or predictor in predicting the response variable or outcome. However, MDA does not provide information about the direction or nature of the relationship between the explanatory variables (geo-factors) and a target variable (landslide). In other words, MDA does not provide insights into the impact of changes in the values of a variable on the probabilistic outcome of the model. Therefore, to understand the marginal effects of geo-factors on the predicted probability of landslides, Partial Dependence Plots (PDP) were computed for each geo-factors. These plots depict the effect of different geo-factors on the probability of landslide occurrences. It is important to note that the marginal effects of geo-factors were computed for the final landslide susceptibility model, i.e., the post-Molave susceptibility model.

**Fig 6:**
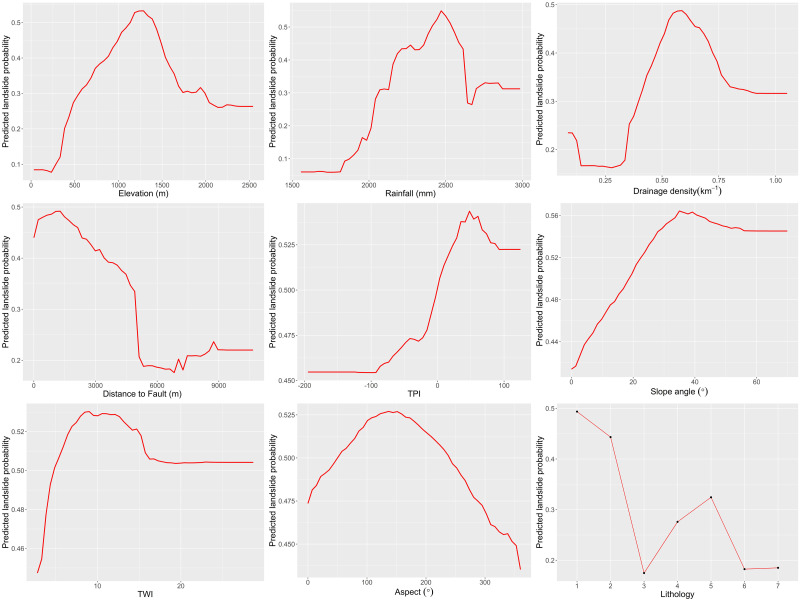
Partial Dependence Plots illustrate variability in predicted landslide probability resulting from changes in the values of individual variables.

Fig 6 illustrates the PDPs that show how the probability of landslides is affected by different values of an individual geo-factor, while all other geo-factors are held constant at their mean. Partial dependence plots were computed for all continuous geo-factors to understand the nonlinear relationship between the values of these geo-factors and change in the probability of landslide occurrence. The predicted probability of landslides gradually increases from 0.05 to 0.5 with increasing elevation, peaking at around 1,250 m, and starts dropping after that. Similarly, the probability of landslides significantly increases with an increase in rainfall amount up to 2,480 mm, then dips sharply between 2,480 mm and 2,650 mm. In contrast, the probability of landslides decreases with an increase in the distance from faults, indicating that areas closer to fault zones will have higher landslide probability. The drainage density seems to have a significant impact, with the probability of landslides initially decreasing, then showing a steady increase to reach the vertex around a drainage density value of 0.58, decreasing gradually as drainage density continues to increase. Similarly, landslide probability increases as TPI values increase, as higher TPI values indicate topographic ridges, which are more prone to landslides than valleys. Moreover, the landslide probability increases from 0.41 to 0.56 as the slope angle steadily increases up to 36°, then landslide probability slightly decreases to 0.54 at the slope angle of 55°, beyond which it remains flat. In contrast, TWI and slope aspects exhibit minimal impact on landslide probability. The importance of a geo-factor in governing landslide probability can also be understood by observing the amount of change in landslide probability on the Y-axis due to changes in the values of a geo-factor on the X-axis. In this regard, elevation and rainfall values exhibit substantial variations in landslide probability range compared to TWI and slope aspects, which display little variation. This observation is further supported by the variable importance graph, where variables showing higher variation in landslide probability, such as elevation and rainfall are ranked higher in the importance graph. In contrast, variables with smaller variations, such as TWI and slope aspects are ranked lower. Lithology was the sole categorical variable incorporated into the model. The highest probability of landslides is observed in Group 1 suites of rocks (Mafic Metamorphic Rocks with quartz-poor component), while the lowest landslide probability was recorded in Group 3 (Igneous- Intrusive Mafic-to-Ultramafic Rocks).

### Model evaluation and uncertainty estimation

**Fig 7:**
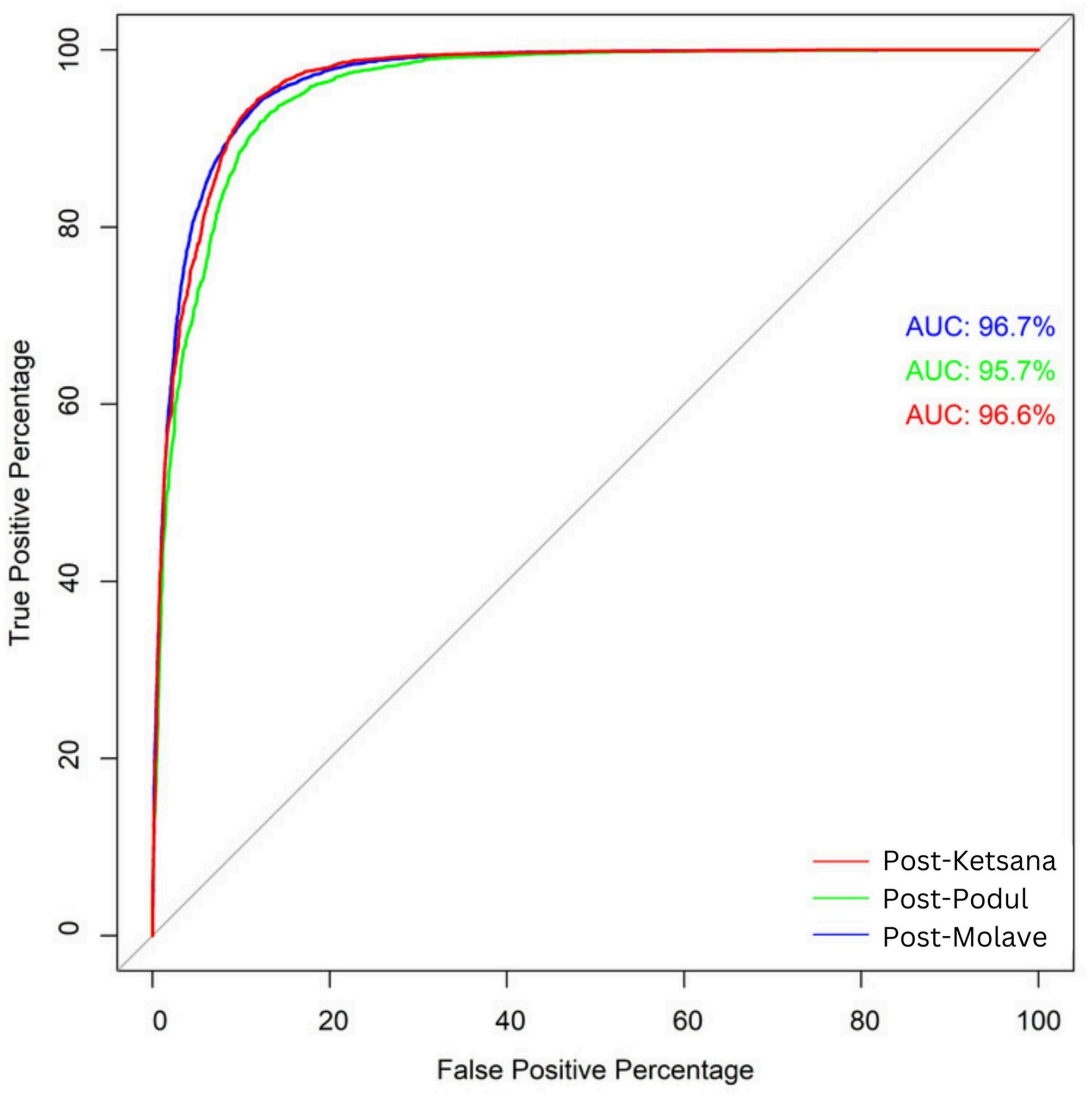
The AUC shows the plot of true positive and false positive percentages of the test data for different RF models. The AUC values of 96.6%, 95.7%, and 96.7% yielded for the post-Ketsana, post-Podul, and post-Molave landslide susceptibility models, respectively.

Table 4 presents the performance metrics of the three landslide susceptibility models developed in this study. The test data reveals high accuracy for all models, with values of 91.30%, 89.82%, and 90.74% for the post-Ketsana, post-Podul, and post-Molave models, respectively. Sensitivity values were also high, with all models achieving greater than 90% sensitivity. Additionally, AUC was calculated for all three models, resulting in values of 96.6%, 95.7%, and 96.7% for the post-Ketsana, post-Podul, and post-Molave models, respectively (Fig 7). These high AUC values indicate the models’ exceptional ability to differentiate between landslide and non-landslide areas, thereby demonstrating the models’ robustness.

**Table 4:** Out-of-bag (OBB) error rates of the RF models and other performance evaluation metrics as calculated for the test data.

Model	Out-of-Bag error rate(%)	Accuracy(%)	Sensitivity(%)	Specificity(%)
Post-Ketsana	6.46	91.30	92.95	89.52
Post-Podul	6.81	89.82	90.87	88.49
Post-Molave	6.82	90.37	90.49	90.24

**Fig 8:**
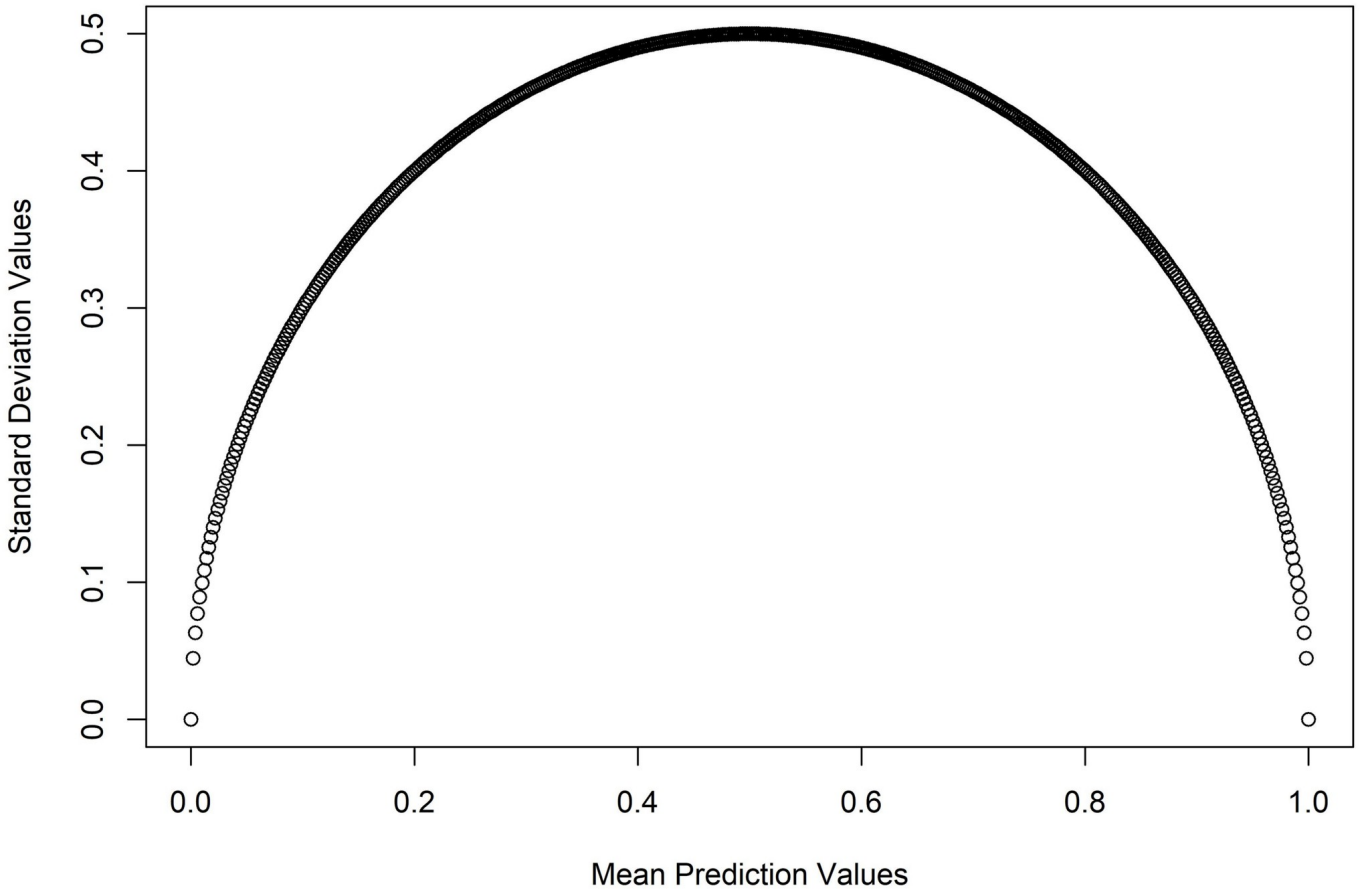
Mean prediction pixel values of RF plotted against the standard deviation of mean pixel values for all individual pixels for the post-Molave landslide susceptibility model. Distribution pattern of mean and standard deviation of the predictions remain same for all landslide susceptibility models i.e., they exhibit a quadratic relationship.

**Fig 9:**
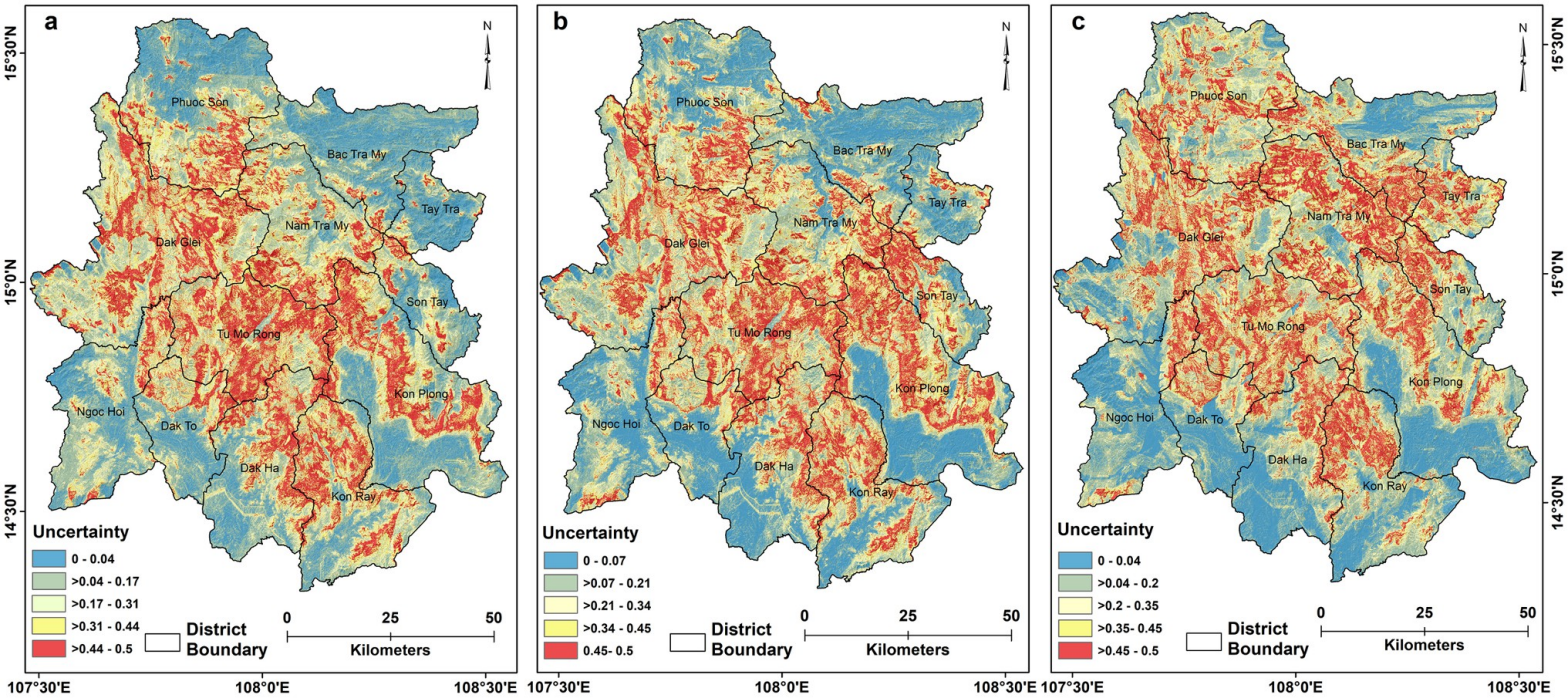
Maps depicting the uncertainty of RF predictions at the pixel level for (a) post-Ketsana, (b) post-Podul, and (c) post-Molave landslide susceptibility models. The uncertainty is estimated by computing the standard deviation of predictions from 500 trees for each pixel across the entire study area. District boundary, https://cmr.earthdata.nasa.gov, https://www.chc.ucsb.edu/data/chirps, Institute of Geological Sciences, (VAST). DOI: 10.6084/m9.figshare.25710606.

The landslide probabilities estimated from the RF models are the mean prediction of the 500 trees for all individual pixels with the outcome of either 0 or 1. The mean prediction values of individual pixels exhibit a quadratic relationship with the standard deviation values of the pixels. The lower the variation in the votes of the trees used to assign a class to a pixel (either landslide or non-landslide), the greater the certainty of accurately classifying the pixel. Therefore, high and low landslide probabilities are associated with low uncertainty values, whereas uncertainty increases as predictive values approach 0.5 and gradually decreases after that (Fig 8). Since the models show variation in landslide susceptibility values for the pixels across time, the spatial distribution of uncertainty value also varies accordingly (Fig 9a–9c). The greatest certainty is found in the north (north of Phouc Son), northeastern (Bac Tra My, Tay Tra districts), southeastern and southwestern (Ngoc Hoi, south of Dak To and Dak Ha districts) portions of the study area that all exhibit low landslide susceptibility. The central part of the study area (including parts of To Mo Rong, Dak Glei, Dak Ha, Kon Plong, Nam Tra My, Son Tay, Phouc Son) shows high uncertainty, which is associated with moderate landslide susceptibility values, and low uncertainties, which are associated with high-to-very high landslide susceptibility values (Fig 9a–9c).

## Discussion

Landslide susceptibility models developed in this study utilized successive landslide inventories to retain the susceptibility information from the previous events, with the final susceptibility model incorporating all three landslide inventories. This methodology was adapted to retain the landslide susceptibility information from the previous events and add that up with the successive model. The landslide susceptibility models predict the spatial probability of landslides assuming that landslides will occur in the future under the influence of the same geo-environmental conditions and triggering factors that initiated them in the past. However, it is important to understand that certain geo-factors are subject to change due to anthropogenic activities or other natural causes. For example, factors like land cover, hydrological conditions, and rainfall patterns may change quickly say in the next 20 years. In this study, average annual rainfall was calculated for different periods and incorporated into the respective landslide susceptibility models as a geo-factor to incorporate the change in the rainfall pattern, if any, in the study area. Moreover, changes in the topographic conditions can occur when a landslide’s source is depleted due to previous landslides or when the slope morphology undergoes alterations and becomes stable. Nevertheless, it is safe to assume that most predisposing factors, such as lithology, geological structure, or topographic position will not change significantly on the time scales relevant to human-landscape interactivity. Some local morphological alterations are possible due to stream erosion, landsliding, and anthropogenic activities like the construction of building pads, roads, and other infrastructures on hillslopes or right at the base of slopes, but widespread morphological changes are foreseeable. Therefore, it is important to update the landslide susceptibility model if and when major landslides occur and/or if any significant modifications affects the predisposing factors, e.g., rapid and extensive deforestation, which would change the hillslope hydrology and likely to decrease the threshold for landslide triggering rainfall.

A RF algorithm was employed to develop three landslide susceptibility models, all of which achieved very high accuracies indicated by their AUC and sensitivity values of the test data (Table 4, Fig 7). While the models achieved reasonably high accuracy, it is important to note that some landslides may have occurred in areas with moderate to low susceptibility, introducing additional uncertainty and error in the susceptibility analysis. Nevertheless, developing landslide prediction models with limited data entail certain elements of uncertainty and error, and the goal should be to reduce the uncertainty and error in the model. This study has attempted to minimize error and uncertainty in the model by deploying a robust machine-learning algorithm and selecting a limited number of explanatory variables to model future landslide susceptibility. The uncertainty of the models was calculated by estimating the standard deviation of the prediction of each tree in the RF model at every pixel. Low model uncertainties are expected in the pixels where the probability of the landslides is either high or low, and model uncertainties are high near the landslide probabilities close to 50 percent. For example, if a pixel has a landslide probability of 0.9 that means 90 percent of the trees in the model classified the pixel as a landslide and only 10 percent classified otherwise. Uncertainty of this pixel will be low since overwhelming numbers of trees identified the pixel as a landslide. on the other hand, if a pixel has a probability of 0.5 that indicates 50 percent of the trees in the model classified that pixel as a landslide, as well as the same number of trees identifying the pixel as a non-landslide. Therefore, uncertainty at this pixel will be very high due to having an equal chance of either landslide or non-landslide classification. Distribution of the mean predictions or probabilities and standard deviations of the pixels constitute a quadratic relation, as observed in Fig 8.

Landslide susceptibility analysis over different periods using multi-temporal inventories of catastrophic landslides revealed a notable transformation in terrain vulnerability to mass wasting. Areas that exhibited marginal susceptibility to landslides in the post-Ketsana period evolved into highly susceptible regions after the post-Molave period. This observation suggests an expansion of landslide-prone areas, signaling a trend likely to persist as more frequent and intense weather events are expected to trigger increasing occurrences of landslides. Perhaps not surprisingly, topographic elevation and annual average rainfall were the two most important geo-factors controlling the occurrences of landslides, as indicated by the variable importance analysis and PDPs. This result suggests that the certain combinations of elevation and rainfall values can strongly influence to increase degree of susceptibility of the topography to landslides. However, analysis of PDPs revealed a decrease in landslide probability beyond certain thresholds of elevation and rainfall. This may be due to the fact that in mountainous regions, like the study area, high rainfall is often associated with high elevation, and it’s possible that the upper reaches of the terrain possess thin soil and rock debris cover due to the factors such as steep slopes and bare rock outcrops. This diminished mass of rock-soil cover could result in a reduced availability of materials capable of initiating landslides, consequently leading to a decrease in landslide probability even in the presence of high precipitation. Lithological composition stood out as the third most important variable for predicting landslides in two out of three susceptibility models. In PDPs, the highest landslide probability was observed in mafic metamorphic lithologies with a quartz-poor component, which includes rock types like biotite schist, sericite schist, biotite granite, two mica granite. These rock types are susceptible to chemical weathering and physical fragmentation, leading to a loss of strength and cohesion, which in turn facilitates mass wasting processes. A prior investigation conducted in Kon Tum province by Bui et al. (2020) identified elevation as the predominant variable influencing their landslide susceptibility model. In another study, Meinhardt et al. (2015) found distance to the road to be the primary determinant in the regions of central Vietnam. Elevation emerged as one of the key variables in our study, consistent with the findings of Bui et al. (2020). Further, the AUC achieved by Bui et al. (2020) and Meinhardt et al. (2015) in their best-performed susceptibility model were 97.3% and 92.3%, respectively, on the test dataset, which is comparable with the current study where AUC ranges between 95.7% and 96.7%. However, it is important to note that both these studies were based on partially completed landslide inventories, with significantly fewer landslide occurrences compared to the dataset utilized in the current study. Furthermore, determining variable importance in these studies employed methodologies different from those in the present study. Therefore, utilization of different landslide datasets and algorithms for landslide susceptibility assessment and computing variable importance, particularly in study areas that do not entirely overlap with the current study, suggests that our findings may not be entirely congruent. It is worth mentioning that the relationship between landslides and geo-factors may not always be linear, or a complex interaction between various geo-factors may initiate landslides; in such cases, the underlying relationship may not always be comprehensible, especially if the study is conducted on a regional-scale. Regional-scale models are designed to reveal the overall trend and variation in landslide susceptibility with an aim towards understanding the general interaction between geo-factors and landslides. However, such models may not always be suitable for understanding the local-scale landslide phenomena. Rather, these regional-scale models can help prioritize areas with high landslide susceptibility for more detailed, site-specific investigations to improve land use planning and hazard mitigation measures.

Understanding the impact of climate change on landslides remains a pivotal area of research focus. However, comprehending the correlation between climate change and landslide occurrences remains challenging for various reasons. The absence of a singular method for analyzing the intricate relationship between landslides and climate, coupled with the incompleteness of historical climate and landslide records limit our ability to assess the influence of future environmental and climate changes on landslide frequency [46, 47]. Additionally, climate and landslide processes operate on distinct geographical and temporal scales, making the reconciliation of these data of divergent scales difficult and uncertain [4]. Therefore, making definitive assertions that climate change will lead to an increase in landslide activity can be erroneous and misleading. However, shifts in hydroclimatic conditions would elicit different responses from various types of slope failures [19]. For instance, the frequency of fast-moving landslides, such as debris flows, is expected to increase with intensified rainfall and wetter hydroclimatic conditions, while decreasing in drier hydroclimatic conditions. Similarly, warmer and drier hydroclimatic conditions could increase the likelihood of wildfires and subsequently, the potential for post-wildfire debris flows. Conversely, the frequency of deep-seated landslides is likely to decrease and their movement rates would decelerate in a warmer and drier world.

Identifying changes in terrain susceptibility to landslides through time-series analysis can be an effective method for monitoring the impact of climate change on landslides at a regional-scale. However, undertaking such an effort requires access to historical landslide information for the region, a resource that is often challenging to obtain, particularly for the Global South. Due to the absence of reliable and complete landslide inventories, this study relied on three completed inventories to perform the spatio-temporal analysis of landslide susceptibility. The availability of more landslide inventories could have helped in performing a higher-frequency temporal analysis, for example, annually or bi-annually, to understand better the subtle changes in landslide susceptibility across the landscape. Therefore, to conduct such high-frequency temporal analyses in the future, records of landslide occurrences must be documented systematically as soon as such information becomes available. Nevertheless, this study has identified a significant escalation in landslide susceptibility across the highlands of central Vietnam from 2009 to 2020. This observation could potentially serve as an early indication of the influence of changing hydroclimatic conditions on landslide occurrences in the region. Moreover, taking into account the tropical climate zone in central Vietnam, where an anticipated rise in the frequency of extreme precipitation events are predicted [4], there is a likelihood of a concomitant increase in landslide activity. The repetitive incidence of such landslides might eventually deplete the materials on slopes, leading to a gradual transformation into a weathering-limited landscape. For instance, it was observed during field investigation that some of the landslides triggered by typhoon Molave had significantly eroded their source materials and no longer has the topsoil covering the slopes. The evacuation of hillslope soils down to bedrock implies that it will require decades, if not centuries, for these landslide slopes to regenerate sediments on their surfaces before experiencing any further mass movement. Considering the possibility of an increase in the frequency of extreme rainfall events in the region, it is crucial to undertake additional investigations aimed at monitoring and recording localized hydroclimatic data alongside landslide activities. The amassed records would serve as a foundation for future research to analyze and establish an empirical relationship between landslide occurrences and hydroclimatic conditions, such as rainfall duration-intensity or antecedent rainfall, aiming to understand the potential impact of climate change on landslide activities in the region, should such a trend exist.

## Supporting information

S1 Fig Drone images of Molave-triggered landslides in central Vietnam (https://doi.org/10.6084/m9.figshare.25723872). (JPG)

S1 Data The folder contains data of landslides triggered by Typhoon Ketsana, Tropical Storm Podul, and Typhoon Molave in shapefile format. (https://doi.org/10.5281/zenodo.11095611). (ZIP)

S2 Data The folder contains “Lithology” and “Distance to fault” data (https://doi.org/10.5281/zenodo.12629901). (ZIP)
