# Supplementary figures and images for: Machine learning-based assessment of regional-scale variation of landslide susceptibility in central Vietnam
Source: PLoS One. 2024 Oct 25;19(10):e0308494. doi: 10.1371/journal.pone.0308494 (PMC11508152; doi:10.1371/journal.pone.0308494)

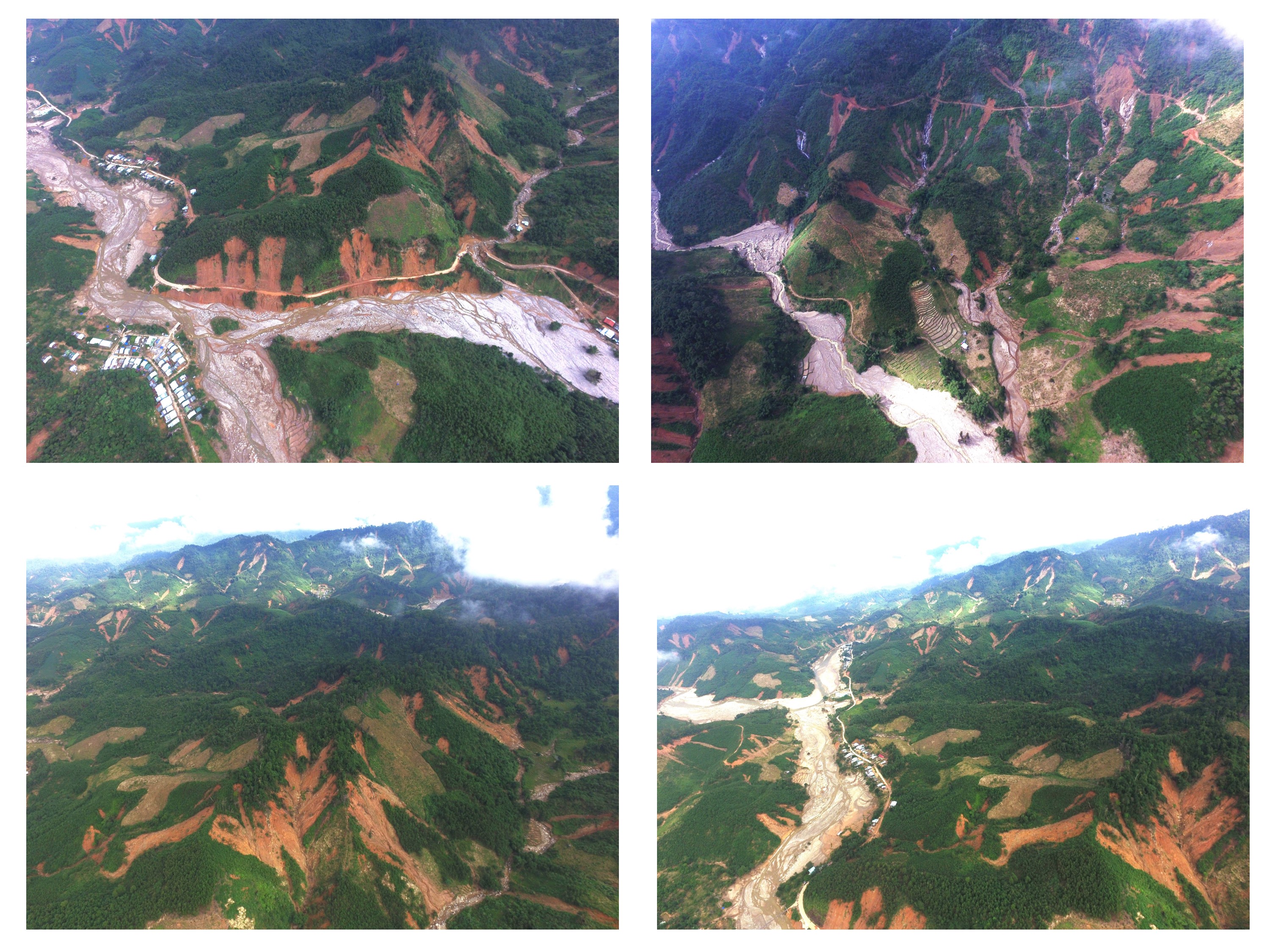

Supplement: S1 Fig — (JPG) [file pone.0308494.s001.jpg]
